# Internet-Based HIV Self-Testing Among Men Who Have Sex With Men Through Pre-exposure Prophylaxis: 3-Month Prospective Cohort Analysis From China

**DOI:** 10.2196/23978

**Published:** 2021-08-27

**Authors:** Jing Zhang, Joseph Tucker, Weiming Tang, Hongyi Wang, Zhenxing Chu, Qinghai Hu, Xiaojie Huang, Yaokai Chen, Hui Wang, Xiaoqing He, Yao Li, Lukun Zhang, Zhili Hu, Rantong Bao, Shangcao Li, Hang Li, Haibo Ding, Yongjun Jiang, Wenqing Geng, Junjie Xu, Hong Shang

**Affiliations:** 1 NHC Key Laboratory of AIDS Immunology National Clinical Research Center for Laboratory Medicine The First Affiliated Hospital of China Medical University Shenyang China; 2 Key Laboratory of AIDS Immunology Chinese Academy of Medical Sciences Shenyang China; 3 Key Laboratory of AIDS Immunology of Liaoning Province Shenyang China; 4 Collaborative Innovation Center for Diagnosis and Treatment of Infectious Diseases Zhejiang University Hangzhou China; 5 Faculty of Infectious and Tropical Diseases London School of Hygiene and Tropical Medicine London United Kingdom; 6 University of North Carolina at Chapel Hill Project-China Guangzhou China; 7 Dermatology Hospital of Southern Medical University Guangzhou China; 8 Center for Infectious Diseases, Beijing YouAn Hospital Capital Medical University Beijing China; 9 Division of Infectious Diseases, Chongqing Public Health Medical Center Chongqing China; 10 Department of Infectious Diseases Shenzhen Third People’s Hospital Shenzhen China

**Keywords:** HIV self-testing, men who have sex with men, pre-exposure prophylaxis, secondary distribution, usage

## Abstract

**Background:**

Routine HIV testing accompanied with pre-exposure prophylaxis (PrEP) requires innovative support in a real-world setting.

**Objective:**

This study aimed to determine the usage of HIV self-testing (HIVST) kits and their secondary distribution to partners among men who have sex with men (MSM) in China, who use PrEP, in an observational study between 2018 and 2019.

**Methods:**

In 4 major cities in China, we prospectively followed-up MSM from the China Real-world oral PrEP demonstration study, which provides daily or on-demand PrEP for 12 months, to assess the usage and secondary distribution of HIVST on quarterly follow-ups. Half of the PrEP users were randomized to receive 2 HIVSTs per month in addition to quarterly facility-based HIV testing. We evaluated the feasibility of providing HIVST to PrEP users.

**Results:**

We recruited 939 MSM and randomized 471 to receive HIVST, among whom 235 (49.9%) were daily and 236 (50.1%) were on-demand PrEP users. At baseline, the median age was 29 years, 390 (82.0%) men had at least college-level education, and 119 (25.3%) had never undergone facility-based HIV testing before. Three months after PrEP initiation, 341 (74.5%) men had used the HIVST provided to them and found it very easy to use. Among them, 180 of 341 (52.8%) men had distributed the HIVST kits it to other MSM, and 132 (51.6%) among the 256 men who returned HIVST results reported that used it with their sexual partners at the onset of intercourse. Participants on daily PrEP were more likely to use HIVST (adjusted hazard ratio=1.3, 95% CI 1.0-1.6) and distribute HIVST kits (adjusted hazard ratio=1.3, 95% CI 1.1-1.7) than those using on-demand PrEP.

**Conclusions:**

MSM who used PrEP had a high rate of usage and secondary distribution of HIVST kits, especially among those on daily PrEP, which suggested high feasibility and necessity for HIVST after PrEP initiation. Assuming that fourth-generation HIVST kits are available, HIVST may be able to replace facility-based HIV testing to a certain extent.

**Trial Registration:**

Chinese Clinical Trial Registry ChiCTR1800020374; https://www.chictr.org.cn/showprojen.aspx?proj=32481

**International Registered Report Identifier (IRRID):**

RR2-10.1136/bmjopen-2019-036231

## Introduction

Oral pre-exposure prophylaxis (PrEP) can reduce HIV infections among adherent men who have sex with men (MSM) [1-3]. While the adherence to PrEP is only approximately 60% in the real-world setting [4], it is crucial for all PrEP users to undergo quarterly HIV testing to avoid a breakthrough infection and acquiring resistance to antiretroviral therapy [5].

However, the implementation of PrEP has increased the rate of missing facility-based testing. Almost 30% of PrEP users did not show up for their first quarterly clinical visits [6], and approximately 40% of them missed their 6-monthly appointment [7]. Difficulty attending clinical visits and missing laboratory testing has become one of the main reasons for the interruption [8] and discontinuation [9] in PrEP use in clinical settings. Since the outbreak of COVID-19, attendance at facility-based HIV testing became more difficult because of clinic closure, challenges with social distancing, and related difficulties [10]. It is crucial to identify strategies to ensure frequent HIV testing among individuals on PrEP.

In order to improve and expand PrEP uptake, nonclinical PrEP approaches are being piloted, such as central dispensing at pharmacies [11], community venues (eg, automated teller machines in shopping malls) [12], schools, and prisons or jails [13]. These approaches still require periodic HIV testing, often through HIV self-testing (HIVST), and be supplemented with internet-based support, which is a convenient and confidential option for HIV testing, allowing people to take an HIV test and learn the outcome in their own home or at other private locations [14], while mailing and application of the test kit, support consultations during testing, uploading of testing outcomes, and follow-ups can be conducted over the internet [15,16].

HIVST can also promote partner testing through secondary distribution of test kits [17]; that is, an individual who is provided multiple self-test kits can distribute them to sexual partners or to others in their social network [18]. Peers within the key HIV-infected population play an important role in facilitating HIV prevention in that population; for example, by promoting the uptake of HIV testing [19] and linkage to care [20]. Providing multiple HIVST kits to PrEP users and encouraging their secondary distribution would potentially empower MSM using PrEP and the promote HIV prevention within the MSM community.

HIVST technology has advanced in recent years. Third-generation HIVST technology has a testing period of 3 weeks, with high sensitivity and specificity [21]. PrEP recipients can easily administer HIVST at home and know their HIV status at least 4-8 weeks ahead of their next quarterly HIV facility-based test, as recommended by World Health Organization and Centers for Disease Control and Prevention [5]. In such a scenario, HIVST can be used to identify a breakthrough infection earlier and prevent 4-8 weeks of antiretroviral therapy in PrEP that could lead to resistance.

We aimed to assess the usage and secondary distribution of internet-based HIVST kits and their correlates among MSM using PrEP, prospectively. This information can provide evidence regarding the utility of HIVST in the era of PrEP and support a better paired and targeted HIV-testing strategy among PrEP users in the future.

## Methods

### Study Design, Setting, and Participants

We conducted a randomized control trial of HIV self-testing among MSM who are PrEP users (Chinese Clinical Trial Registry# ChiCTR1800020374). Participants were recruited from among MSM in the ongoing PrEP pragmatic trial (CROPrEP, ChiCTR-IIN-17013762), which provides regimens of daily and on-demand PrEP, in 4 major Chinese cities (Beijing, Shenyang, Chongqing, and Shenzhen) from December 2018 to September 2019. CROPrEP provides all participants standard care of PrEP, including quarterly facility-based HIV testing. Given the well-documented protective effect of PrEP, the sample size of the CROPrEP study at each site was not determined through power calculations. The sample size at each site was increased maximally by fully considering the human resources and capability of each study site [22].

We randomized half of the enrolled PrEP recipients to provide them 2 HIV self-tests per month, in addition to the standard care. The study team has generated a service account on one of the most popular social media in China (WeChat) to provide web-based services on the application of extra testing kits, instructions on self-testing, real-time consultation with the staff, uploading of test outcomes, and follow-up questionnaires. All HIVST kits distributed or secondarily distributed were marked with a serial number. Participants who received HIVST were also encouraged to share them with their male sexual partners. All participants were prospectively followed up quarterly. This study adhered to the STROBE (Strengthening the Reporting of Observational studies in Epidemiology) checklist (Multimedia Appendix 1).

The study sites are general hospitals equipped with HIV voluntary counseling and testing clinics and HIV treatment clinics with physicians specialized in infectious diseases in 4 major cities in China: The Youan Hospital of Capital Medical University, Beijing; The First Affiliated Hospital of China Medical University, Shenyang; The Chongqing Public Health Medical Center of Southwest University, Chongqing; and The Third People’s Hospital, Shenzhen.

Participants were eligible to participate in this study if they (1) were designated male at birth; (2) have sex with male partners; (3) are aged 18-65 years; (4) report having ≥1 of the following risk factors in the last 6 months: (a) had condomless receptive anal sex with a male partner, (b) had more than 2 male sexual partners, (c) had self-reported sexually transmitted infections (STIs) such as syphilis, gonorrhea, chlamydia, chancroid, or lymphogranuloma venereum, or (d) have ever used postexposure prophylaxis medication, but have not received postexposure prophylaxis medication in the previous month; (5) have a nonreactive outcome on a fourth-generation HIV enzyme-linked immunosorbent assay test at baseline screening and undetectable HIV-1 RNA; (6) have no evidence of severe liver or kidney dysfunction on a comprehensive evaluation (including physical examination, urine test, and blood biochemical examination); and (7) indicate willingness to participate and sign an informed consent form. Individuals were excluded if they (1) are deemed ineligible on eligibility evaluation for the CROPrEP trial, (2) refuse to accept or use the HIVST kits (with reasons recorded), or (3) refuse to sign the informed consent form.

To prevent loss to follow-up bias, the clinicians and study staff provided one-on-one personalized compliance support, counseling, and cohort maintenance during the follow-up period. Furthermore, community-based organizations and the weekly internet-based retention strategies were used to strengthen group- and individual-level supervision of retention and cohort management. This internet-based strategy included interactive peer counseling focusing on study retention; a short message providing routine follow-up visit reminders, along with a live chat.

### Measures

Information on demographic characteristics (including age, education level, marital status, and monthly income), HIV-related risk behavior in the past 3 months (frequency of anal intercourse, and instances of condomless anal intercourse, substance abuse, etc), and testing history for HIV and STIs in the past were collected at baseline through self-administrated questionnaires distributed on each participants’ smartphone. The aforementioned information was obtained in accordance with a systematic review that analyzed studies on HIV testing behaviors among MSM in China [23]. Participants were grouped into <25-year-old and ≥25-year-old age groups in accordance with the definition of youth of the United Nations [24]. Participants were also grouped by their median monthly income. Measures of monthly income were separated by the median income. Usage of HIVST and secondary distribution of HIVST to male sexual partners and other MSM were recorded at quarterly follow-up among all participants through self-administrated questionnaires on each participants’ smartphone. The returned results of photographs of used HIVST were uploaded by participants after they used HIVST kits, and information regarding the place, occasion, and ease of using HIVST was collected. The proportion of HIVST usage was calculated among participants retained at the third month follow-up visit. Furthermore, the proportion of HIVST secondary distribution was calculated among participants who had self-reported usage of HIVST at the third month follow-up visit.

### Statistical Analyses

Statistical analysis was conducted using SPSS (version 20.0, IBM Corp). Demographics, the PrEP regimen, and HIV-related risk behavior in the past 3 months among all participants were expressed as numbers and percentages. Follow-up time was determined as the number of days from the date of enrollment to the date of quarterly follow-up. Univariable and multivariable Cox regression analyses were performed to assess the predictors of self-reported usage of HIVST and secondary distribution of HIVST at the first quarterly follow-up. Variables with *P*<.20 on univariable analysis were included in the multivariable model to avoid the omission of clinically relevant variables, which had an underestimated effect in univariable analysis [25,26]. Variables in the final model were selected with a forward stepwise procedure. Hazard ratios (HRs) and adjusted HRs (aHRs) were calculated. On multivariate analysis, *P*<.05 was considered the cut-off for a significant difference. We plotted Kaplan–Meier survival curves for predictors for HIVST usage and their secondary distribution during multivariable Cox regression analysis with *P*<.05 indicating significance.

### Ethics Approval

The study protocol was approved by the ethics review board of the First Affiliated Hospital of China Medical University (IRB-2018-273), Shenyang. Written informed consent was obtained from each participant before collecting study information or blood samples. Participants voluntarily participated in the study and had the right to refuse to answer any question. Participants had the right to withdraw from the study without penalty. The protocols for the CROPrEP trial [25] and this study [27] have been published. This HIVST study among PrEP recipients was registered on the Chinese Clinical Trial Registry (trial ID ChiCTR1800020374).

## Results

### Baseline Demographics and Behavioral Characteristics

A total of 939 MSM were recruited from the CROPrEP trial, with 470 daily PrEP users and 469 on-demand PrEP users. In total, 471 men were randomized to receive 2 HIV self-tests per month, in addition to the standard care, which included 235 (49.0%) daily PrEP users and 236 (50.1%) on-demand PrEP users.

The median age of these 471 participants at baseline was 29 years (quantile 25-35 years); among them, 390 (82.0%) had an education level of undergraduate and above, 241 (51.2%) had a monthly income of less than US $857, and 266 (56.5%) were single. Regarding HIV-related risk behavior in the past 3 months, 165 (35.0%) individuals had had anal intercourse every week and 234 (49.7%) had had anal intercourse every month, 312 (66.2%) of them had condomless anal intercourse with male sexual partners, 226 (48.0%) used Poppers in the previous 3 months, and 103 (22.5%) had participated in group sex. In total, 39 (8.3%) of them self-reported having STI-related symptoms in the previous 12 months. Further, 375 (79.6%) participants self-reported having used HIVST in the past, and 352 (74.7%) reported having undergone facility-based HIV testing in the past (Table 1).

**Table 1:** Baseline characteristics of pre-exposure prophylaxis recipients carrying out HIV self-testing in 4 major cities of China (N=471).

Variable		Participants, n (%)
**Pre-exposure prophylaxis regimen**		
	Daily	235 (49.9)
	On-demand	236 (50.1)
**Age (median 29.0 years, quantiles 25.0-35.0 years)**		
	<25	84 (17.8)
	≥25	387 (82.2)
**Education level**		
	Senior high and below	81 (17.2)
	Undergraduate and above	390 (82.8)
**Monthly income (US $)**		
	≤857	241 (51.2)
	≥858	230 (48.8)
**Marital status**		
	Single	266 (56.5)
	Married or cohabiting with a female	30 (6.4)
	In a relationship or cohabitating with a male	175 (37.2)
**Frequency of anal intercourse in the past 3 months**		
	Every day	8 (1.7)
	Every week	165 (35.0)
	Every month	234 (49.7)
	Less than once a month	64 (13.6)
**Had condomless anal intercourse in the previous 3 months**		
	Yes	312 (66.2)
	No	159 (33.8)
**Had used Poppers in the previous 3 months**		
	Yes	226 (48.0)
	No	245 (52.0)
**Had group sex in the previous 3 months^a^**		
	Yes	103 (22.5)
	No	354 (77.5)
**Had sexually transmitted infection–related symptoms in the previous 12 months**		
	Yes	39 (8.3)
	No	432 (91.7)
**Have ever used HIV self-testing in the past**		
	Yes	375 (79.6)
	No	96 (20.4)
**Have ever undergone facility-based HIV testing in the past**		
	Yes	352 (74.7)
	No	119 (25.3)

^a^There were 14 missing data.

### Usage, Returning Results, and Sharing of HIVST at the First Quarterly Follow-ups

**Figure 1:**
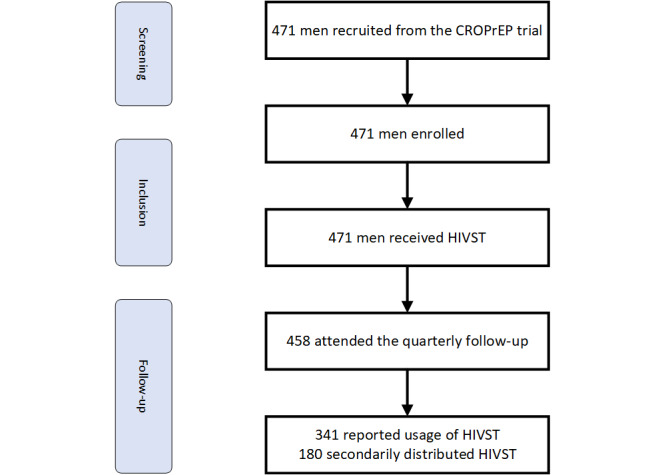
Study flow diagram. HIVST: HIV self-testing.

Among these 471 men, 458 men were retained at the first quarterly follow-up, resulting in a retention rate of 97.2%. At the first quarterly of PrEP initiation, a total of 341 (74.5%) participants self-reported that they had used the HIVST in the past 3 months, among whom 256 (75.1%) returned HIVST testing outcomes by uploading photographs of the used HIVST kits marked with a serial number. In total, 260 (76.2%) men self-reported that they had recommended HIVST to their male sexual partners or gay friends, and 180 (52.8%) participants self-reported that they had shared their HIVST kits with the male sexual partners or gay friends. Among the 256 participants who had returned HIVST outcomes, the most frequent place of using HIVST was at home (n=218, 85.2%). In total, 132 (51.6%) men reported that HIVST was used immediately before or after sexual intercourse with their male sexual partners (Figure 1).

### Correlation of Self-Reported Usage of HIVST on Quarterly Follow-up

On univariable Cox regression analysis, men with the following variables were more likely to use HIVST during follow-up: being on daily PrEP (vs on-demand PrEP, HR=1.273, 95% CI 1.028-1.576; *P*=.03), having an education level of senior high or below (vs college and above, HR=1.393, 95% CI 1.054-1.841; *P*=.02), having STI-related symptoms in the previous 12 months (vs not having symptoms, HR=1.384, 95% CI 0.956-2.004; *P*=.09), having ever used HIVST in the past (vs having used HIVST in the past, HR=1.492, 95% CI 1.102-2.020; *P*=.01). Participants with the following variables were less likely to use HIVST: having had anal intercourse every day in the past 3 months (vs less than once a month, HR=0.396, 95% CI 0.143-1.101; *P*=.08), having had condomless anal intercourse in the past 3 months (vs not having had condomless anal intercourse, HR=0.825, 95% CI 0.687-0.990; *P*=.04) (Table 2).

**Figure 2:**
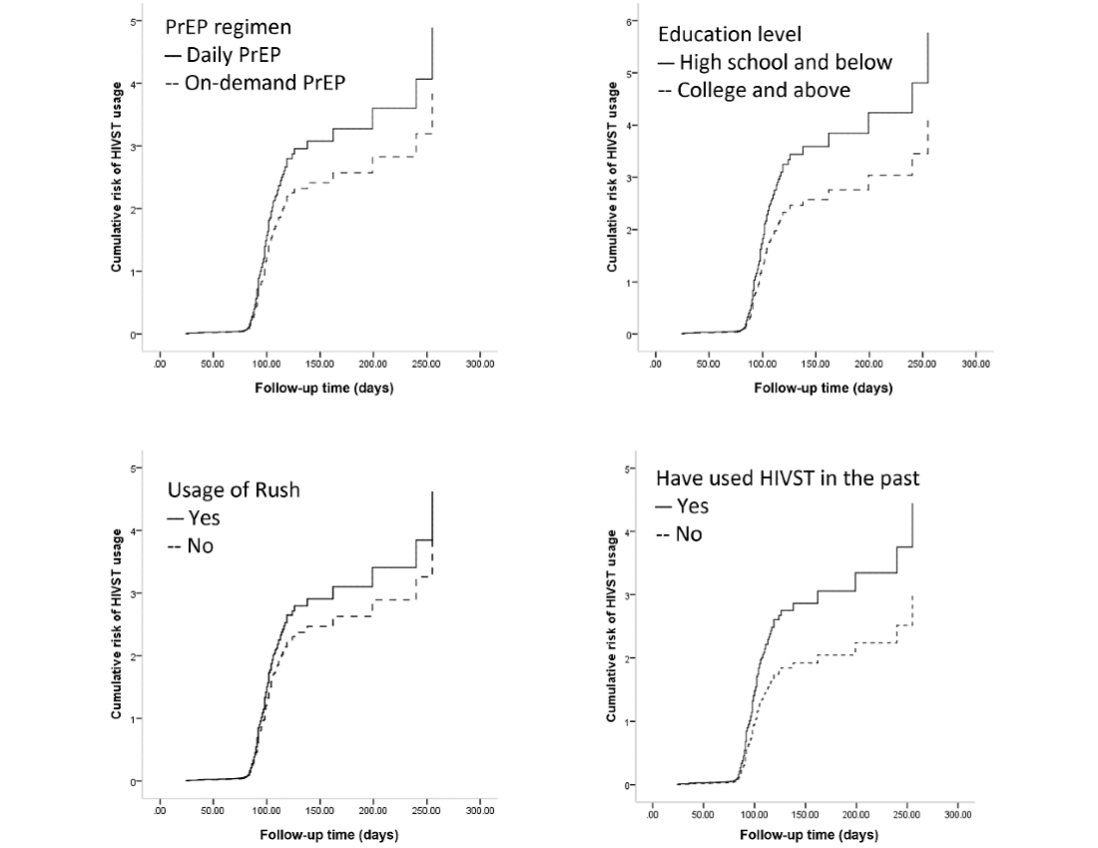
Kaplan–Meier survival curves for predictors of HIVST usage. HIVST: HIV self-testing, PrEP: pre-exposure prophylaxis.

After the forward stepwise procedure, all variables with *P*<.20 on univariable regression analysis were included in the multivariable regression model, and participants with the following variables were more likely to use HIVST during follow-ups: being on daily PrEP (vs on-demand PrEP, aHR=1.298, 95% CI 1.047-1.608; *P*=.02), having an education level of senior high or below (vs undergraduate and above, aHR=1.482, 95% CI 1.119-1.964; *P*=.006), and having used HIVST in the past (vs not having used HIVST in the past, aHR=1.642, 95% CI 1.205-2.242; *P*=.002). Furthermore, participants who self-reported having used Poppers in the past 3 months (vs those not having used Poppers in the past 3 months, aHR=0.788, 95% CI 0.633-9.981; *P*=.03) were less likely to use HIVST (Table 2 and Figure 2).

**Table 2:** Cox regression model of correlates of usage of HIV self-testing among pre-exposure prophylaxis recipients within 3 months of treatment initiation in 4 major cities in China (N=471).

Variable		Usage of HIV self-testing				Secondary distribution of HIV self-test kits			
		Hazard ratio (95% CI)	*P* value	Adjusted hazard ratio (95% CI)^a^	*P* value	Hazard ratio (95% CI)	*P* value	Adjusted hazard ratio (95% CI)	*P* value
Pre-exposure prophylaxis regimen of daily (vs on-demand) users		1.273 (1.028-1.576)	.03	1.298 (1.047-1.608)	.02	1.343 (1.072-1.682)	.01	1.352 (1.079-1.694)	.009
Age<25 years		0.990 (0.751-1.307)	.95	—^b^	—	1.066 (0.739-1.539)	.73	—	—
Education level of high school and below		1.393 (1.054-1.841)	.02	1.482 (1.119-1.964)	.006	1.151 (0.840-1.578)	.38	—	—
Monthly income <US $857		1.149 (0.928-1.423)	.20	—	—	1.074 (0.858-1.346)	.53	—	—
Currently married (vs single)		1.436 (0.811-2.263)	.12	—	—	1.523 (0.963-2.408)	.07	1.706 (1.074-2.709)	.02
**Frequency of anal intercourse in the previous 3 months**									
	Every day	1.0	Reference	—	—	1.0	Reference	—	—
	Every week	1.969 (0.726-5.342)	.18	—	—	1.480 (0.603-3.634)	.39	—	—
	Every month	2.102 (0.779-5.673)	.14	—	—	1.511 (0.619-3.688)	.36	—	—
	Less than once a month	2.523 (0.909-7.004)	.08	—	—	1.481 (0.580-3.783)	.41	—	—
Had condomless anal intercourse in the past 3 months		0.825 (0.687-0.990)	.04	—	—	—	.33	—	—
Used Poppers in the past 3 months		0.848 (0.684-1.049)	.13	0.788 (0.633-0.981)	.03	0.953 (0.761-1.193)	.68	—	—
Had group sex in the past 3 months		1.011 (0.781-1.310)	.93	—	—	0.948 (0.724-1.241)	.70	—	—
Had sexually transmitted infection–related symptoms in the past 12 months		1.384 (0.956-2.004)	.09	—	—	1.154 (0.760-1.753)	.50	—	—
Have performed HIV self-testing in the past		1.492 (1.102-2.020)	.01	1.643 (1.205-2.242)	.002	1.415 (1.034-1.937)	.03	1.464 (1.066-2.009)	.02
Have undergone facility-based HIV testing in the past		1.166 (0.904-1.503)	.24	—	—	1.131 (0.867-1.476)	.36	—	—

^a^Variables with *P*<.20 on univariable analysis were included in the multivariable Cox regression model.

^b^—: not determined.

### Correlation of Secondary Distribution of HIVST Kits at Quarterly Follow-up

On univariable Cox regression analysis, participants with the following variables were more likely to secondarily distribute HIVST kits during follow-up: being on daily PrEP (vs on-demand PrEP, HR=1.343, 95% CI 1.072-1.682; *P*=.01), being currently married (vs being single, HR=1.523, 95% CI 0.963-2.408; *P*=.07), having ever used HIVST in the past (vs having never used HIVST in the past, HR=1.415, 95% CI 1.034-1.937; *P*=.03) (Table 2).

**Figure 3:**
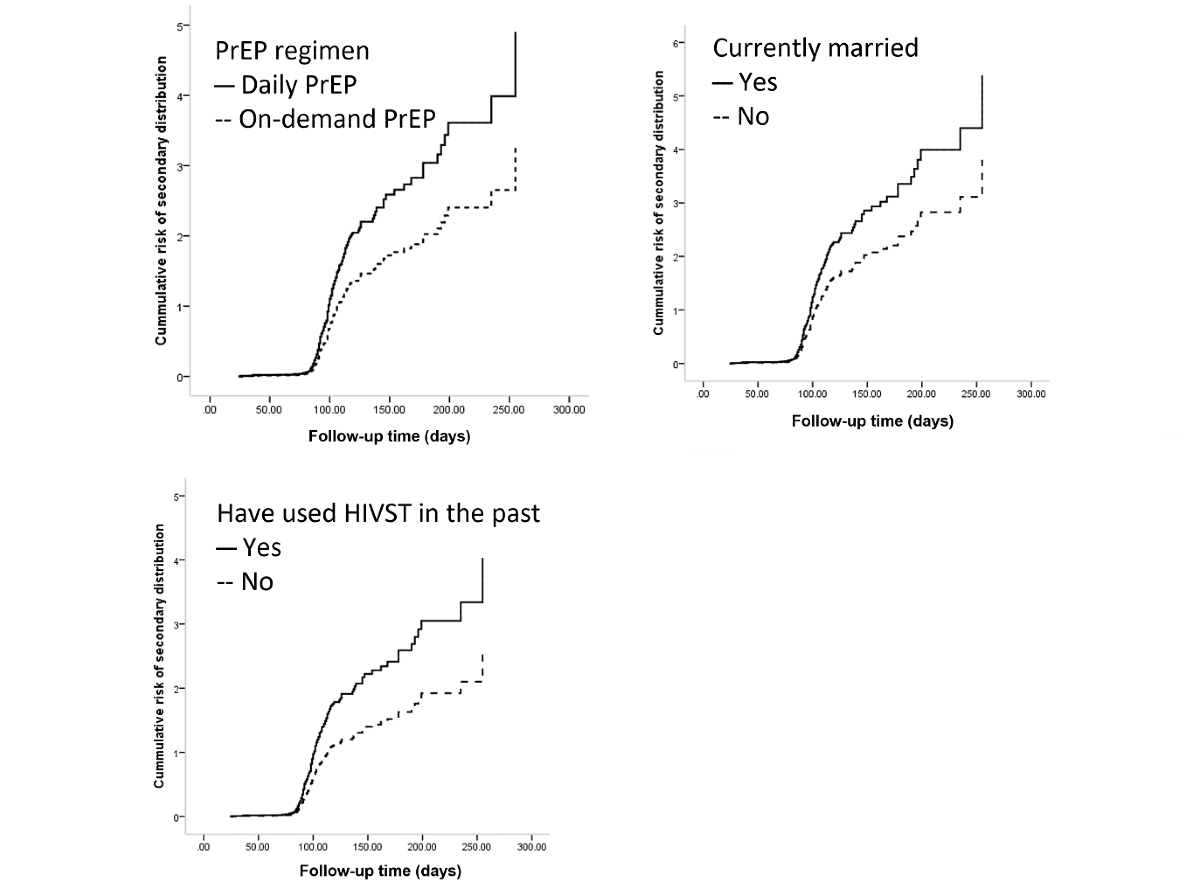
Kaplan–Meier survival curves for predictors of HIVST secondary distribution. HIVST: HIV self-testing; PrEP: pre-exposure prophylaxis.

After the stepwise forward procedure, all variables with *P*<.20 on univariable regression were included in the multivariable regression model, and participants with following variables were more likely to secondarily distribute HIVST during follow-ups: being on daily PrEP (vs on-demand PrEP, aHR=1.352, 95% CI 1.079-1.694; *P*=.009), being currently married (vs single, aHR=1.706, 95% CI 1.074-2.709; *P*=.02), and having used HIVST in the past (vs not having used HIVST in the past, aHR=1.464, 95% CI 1.066-2.009 *P*=.02) (Figure 3).

## Discussion

### Principal Findings

We prospectively identified a high rate of usage and secondary distribution of the internet-based HIVST among MSM who are using PrEP in a multicenter pragmatic trial in China, especially among the daily PrEP users. This result warrants the internet-based HIVST as a crucial part of the HIV testing strategy paired with PrEP among the MSM population. Furthermore, the high rate of secondary distribution of HIVST kits and self-testing among sexual partners among PrEP users assures a promising future for peer-initiated expanded HIV testing and maximization of the HIV prevention among the MSM community.

Approximately 80% of PrEP users have used HIVST within 3 months after PrEP initiation and found it easy to use, reflecting HIV testing behavior among PrEP recipients. Every 4 of 5 of these PrEP users had used HIVST before the initiation of PrEP, which is much higher than the level among the general MSM population in China (20%-40%) [28,29]. Since difficulty attending facility-based testing was one of the main reasons for discontinuing PrEP during implementation [8,9], this high usage of HIVST among PrEP users warrants future consideration of substituting some facility-based HIV testing during PrEP and provides evidence in support of HIVST to support PrEP users during the COVID-19 pandemic in the future, since facility-based services and in-person patient-clinician contact has been limited because of the pandemic [30].

Notably, there was a high rate of secondary distribution of HIVST and testing of sexual partners among those who had used HIVST after PrEP initiation. Half of the HIVST was used to test together with their sexual partners immediately before or after intercourse. This is significant because of the limited success and high cost of accessing MSM at clinics or provider-initiated expanded HIV testing strategies [31,32]. According to previous studies, MSM who accept or uptake PrEP were more concerned about their partners’ HIV status [33] and had more sexual partners [34], which makes them excellent facilitators for expanded HIV testing among the MSM population. Empowering them with HIVST as a tool to undertake secondary distribution and partner testing can maximize the impact of PrEP on the MSM community via peer-initiated HIV prevention campaigns. Future studies should vigorously pursue and explore the critical role of PrEP users in HIV prevention within the key populations.

Factors promoting the usage and secondary distribution of HIVST among PrEP users were also identified. Men on daily PrEP were more likely to use and secondarily distribute HIVST after PrEP initiation than those on the on-demand regimen. Since most of the HIVST was used immediately before or after sexual intercourse, this correlation can be explained by the fact that MSM on daily PrEP displayed more frequent HIV-related risk behavior and a higher number of sexual partners [35,36]. According to HIV transmission network studies, MSM with a higher number of sexual partners are associated more firmly with other MSM and had a crucial impact on the network of HIV transmission within the population. This correlation illustrates that MSM on daily PrEP play a more critical role in expanded HIV testing and HIV prevention campaigns as peers among the MSM community. Studies on the social network of PrEP recipients built through HIVST distribution should be actively pursued in the future.

An inhibitor of the use of HIVST among PrEP users is the use of Poppers, which is one of the most popular recreational drugs among MSM worldwide [37]. According to previous studies, Poppers users are more likely to consider themselves PrEP candidates and more likely to be a current PrEP user [38]. However, Poppers usage among MSM is associated with an increased risk of HIV infection [39,40] and a higher probability of participating in group sex [40], which indicates the necessity of timely HIV testing. This inhibitor should draw attention from PrEP providers to address timely HIV testing for PrEP users.

### Limitations

This study has several limitations. First, there is no fourth-generation HIVST kit available currently in China. These are available in the United Kingdom and in other countries and would potentially allow this model to replace some of the facility-based testing. Second, this study was conducted before the COVID-19 pandemic, which might underestimate the usage of HIVST among PrEP users. Third, this study only assessed the usage and secondary distribution within the first 3 months after PrEP initiation, which requires a longer follow-up period to illustrate its long-term usage. Finally, this study only included participants from 4 major cities in China, which did not represent the general MSM population in China.

### Conclusions

Within a pragmatic setting, MSM who are using PrEP had a high rate of usage and secondary distribution of HIVST kits, especially among those on daily PrEP. Offering multiple HIVST to PrEP users to replace some of the facility-based HIV testing and to facilitate expanded HIV testing is feasible and necessary, especially during the COVID-19 pandemic.

## Appendix

Multimedia Appendix 1 STROBE checklist.

## Abbreviations

aHR: adjusted hazard ratio
HIVST: HIV self-testing
HR: hazard ratio
PrEP: pre-exposure prophylaxis
STI: sexually transmitted infection
